# Development of an ultrasound-guided radiofrequency ablation technique in the equine cadaveric distal limb: histological findings and potential for treating chronic lameness

**DOI:** 10.3389/fvets.2024.1437989

**Published:** 2024-08-23

**Authors:** Martina Amari, Vanessa Rabbogliatti, Giuliano Ravasio, Luigi Auletta, Federica Alessandra Brioschi, Pietro Riccaboni, Silvia Dell’Aere, Paola Roccabianca

**Affiliations:** Department of Veterinary Medicine and Animal Sciences, University of Milan, Lodi, Italy

**Keywords:** axonal thermal damage, chronic pain, horses, palmar digital nerves, thermal radiofrequency, histopathological nerve lesion

## Abstract

**Introduction:**

Radiofrequency (RF) relieves chronic pain in humans, but it is unexplored in horses affected by chronic lameness. This study aims to describe the technique and the histological effects of ultrasound (US)-guided radiofrequency ablation (RFA) of palmar digital nerves (PDNs) in horse’s fetlock and pastern, *ex vivo*.

**Methods:**

After assessing the US anatomy of lateral and medial PDNs in fetlock and pastern *in vivo* (*n* = 10 horses; 20 forelimbs), US-guided RFA was performed on these sites in cadaveric forelimbs (*n* = 10) applying four different settings with increasing invasiveness (*n* = 40 total treatments): 60°C, 6 min (GROUP LOW); 70°C, 4 min (GROUP MEDIUM); 90°C, 2 min (GROUP HIGH); 80°C, 8 min (GROUP VERY HIGH). Needle-tip-to-nerve proximity was assessed with US and methylene blue, injected through the port of the RF needle. Nerves were collected for microscopical assessment.

**Results:**

Transverse palmaro-lateral and palmaro-medial US images of fetlock and pastern detected PDNs consistently, close to the palmar digital artery. With in-plane US technique, RFA was performed at target in 31/40 cases, with significantly higher number of failures in fetlock (*p* = 0.008). PDNs histology identified thermal injury/coagulation with axonal degeneration and collagen homogenation. Nuclear smearing of arterial leyomyocytes was also observed. Nerve coagulation was significantly associated with treatment (*p* = 0.03) and needle-tip-to-nerve proximity (US distance: *p* = 0.009; blue distance: *p* = 0.04).

**Discussion:**

The PDNs were easily visualized and reached with the RF needle by US in-plane-guided technique. RFA produced axonal thermal damage and intensity-related coagulation effectiveness. To ensure effective nerve coagulation, it is crucial that the needle is accurately positioned in close proximity to the target nerve. Based on the histopathological findings, HIGH and VERY HIGH RFA treatments might be worth of being tested *in vivo* in clinical studies aimed at treating chronic lameness of the distal forelimb in horses.

## 1 Introduction

Chronic lameness is a major cause of reduced life quality in horses (1). Limited analgesic options and prolonged confinement during convalescence may lead to consider euthanasia (2, 3). The use of systemic non-steroidal anti-inflammatory drugs provides some degree of relief but long-term administration is often required (4). Moreover, several adverse effects including gastrointestinal tract ulceration, right dorsal colitis, and/or acute nephrotoxicity are reported when non-steroidal anti-inflammatory drugs are administered for prolonged time (5). In addition, horses suffering from neuropathic pain may not respond favorably to non-steroidal anti-inflammatory drugs (6) due to maladaptive sensitization of pain pathways, resulting in ongoing pain, hyperalgesia and allodynia (1). Palmar and plantar digital surgical neurectomy may also be an option when other alternatives are not available, but it may lead to complications such as painful neuroma formation (7, 8) and an increased risks of injury to the lower limb (9).

Radiofrequency (RF) is a non-pharmacological interventional technique that has been applied to treat neuropathic chronic pain unresponsive to other pharmacological and non-pharmacological techniques in humans (10–12). The duration of pain relief after RF varies from 3 to 24 months, depending on the treated site, the technique used, and the individualized response (13–16). This therapy and its efficacy seem not to have been evaluated in horses. The RF entails placing an insulated needle with a conductive tip close to the target nerve. A high-frequency electric current generator, to which the needle is connected, produces a small electric field at the tip, generating thermal energy that creates a small lesion near the affected target site. Radiofrequency ablation (RFA) employs temperature ranges of 60–90°C to induce thermal neurodestructive lesions (17) that result in Wallerian degeneration developing over the next 2–3 weeks. These changes cause subsequent alteration of the transmission and conduction of nociceptive impulses and interruption of pain signals (18, 19). Since the application of RFA induces, even if temporary (17), denervation, it is generally limited to nerves composed by sensory fibers only, as a motor deficit could ensue (19). The equine distal forelimb is innervated by the medial and lateral palmar nerves, which become the medial and lateral palmar digital nerves (PDNs) proximal to the metacarpophalangeal joint (20). At this level, nerves do not contain myelinated motor fibers and are mainly composed of myelinated sensory fibers, unmyelinated sympathetic and unmyelinated peptidergic sensory axons (21). Therefore, RFA may represent an attractive therapeutic option in horses with chronic lameness unresponsive to other treatments. However, the occurrence of complications similar to those described following surgical neurectomy cannot be excluded. The aims of the present study were to: (1) Identify the ultrasonographic anatomical landmarks of the lateral and medial PDNs in the fetlock and pastern regions in living horses; (2) Evaluate the feasibility of close RF needle-to-nerve positioning in these two regions using an ultrasound (US)-guided technique, *ex vivo*; (3) Describe the histopathological nerve lesions produced by RFA and evaluate the frequency of nerve coagulation at different settings with increasing invasiveness. The hypotheses were that the nerves could be easily visualized and reached with the needle by US-guided technique, and that the coagulative effectiveness would increase as the intensity of the RFA treatment performed increased.

## 2 Materials and methods

The present study complied with ethical standards, and it was conducted under the approval of the Institutional Ethical Committee for Animal Care at the University of Milan (OPBA_51_2023). Informed written consent was obtained by the owners of all live horses enrolled in the study. No Ethical Committee for Animal Care oversight was required in the *ex vivo* phases, conducted using material collected during *post-mortem* examination after collection of owners’ written consent. The study was divided into three phases: phase I, concerning the assessment and description of the US anatomy of the lateral and medial PDNs in the region of the pastern and fetlock of live horses, with a specific focus on the US landmarks for effective US-guided RF needle positioning; phase II, consisting in the application of the US-guided RFA treatment and the injection of a small volume of methylene blue solution into isolated equine distal forelimbs, and the subsequent anatomical dissection to assess needle tip-to-nerve proximity; phase III, evaluating histological nerve lesions produced by four RFA treatment protocols on the forelimbs from phase II.

### 2.1 Phase I. Ultrasonographic landmarks of PDNs in fetlock and pastern regions: *in vivo* study

**Figure 1:**
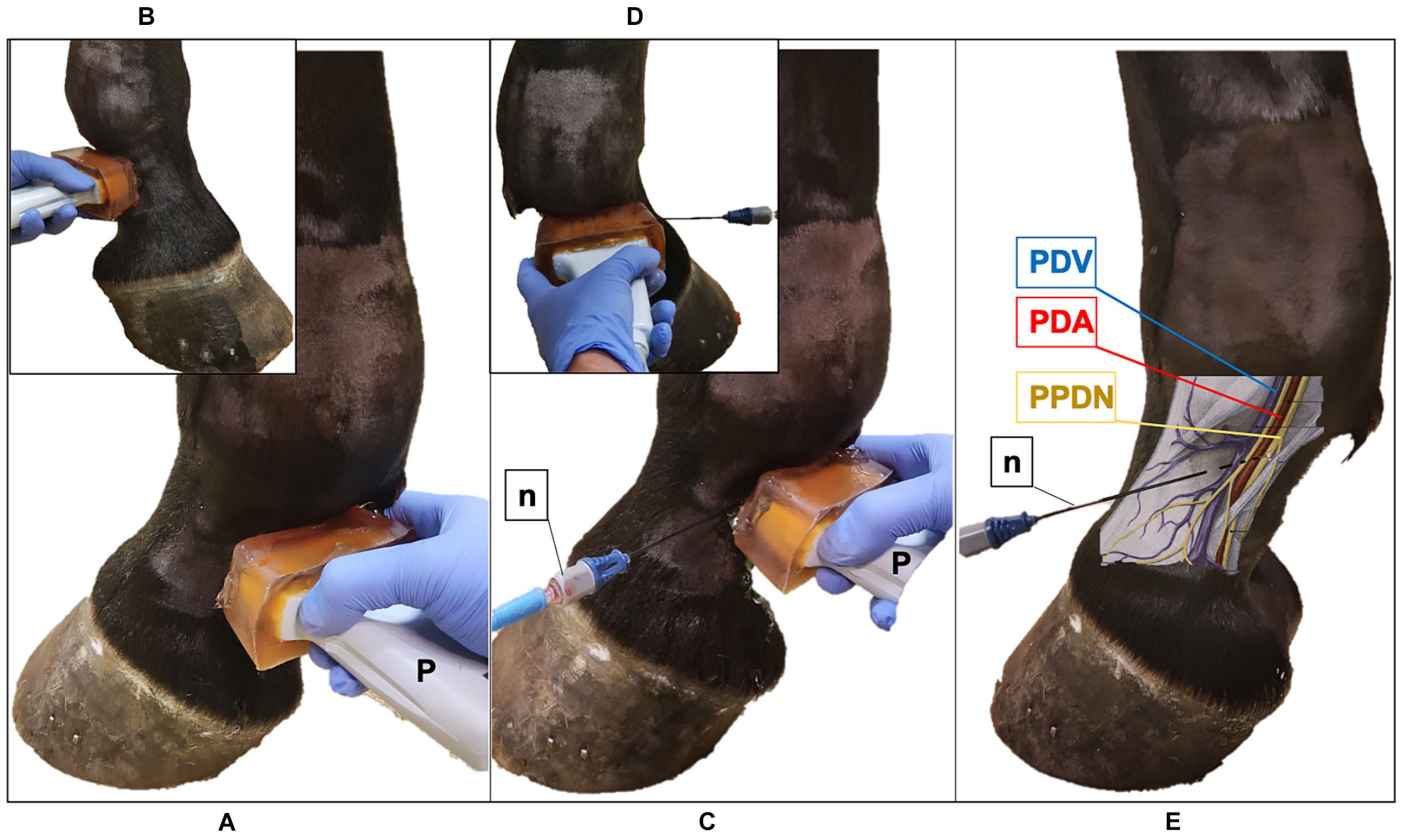
Ultrasound (US) probe and the radiofrequency (RF) needle positioning in the pastern region of the equine forelimb. **(A)** The US probe (P) is positioned transversal to the middle third of the proximal phalanx, with the marker pointing dorsally; palmaro-medial approach. **(B)** Palmaro-lateral approach. **(C)** US-guided in-plane technique, palmaro-medial approach: the RF needle (n) is inserted with a dorso-palmar direction to reach the proper palmar digital nerve (PPDN). **(D)** Palmaro-lateral approach. **(E)** Anatomical representation of the neurovascular bundle: RF needle reaches the PPDN in the subcutaneous tissue. PDV, palmar digital vein; PDA, palmar digital artery.

**Figure 2:**
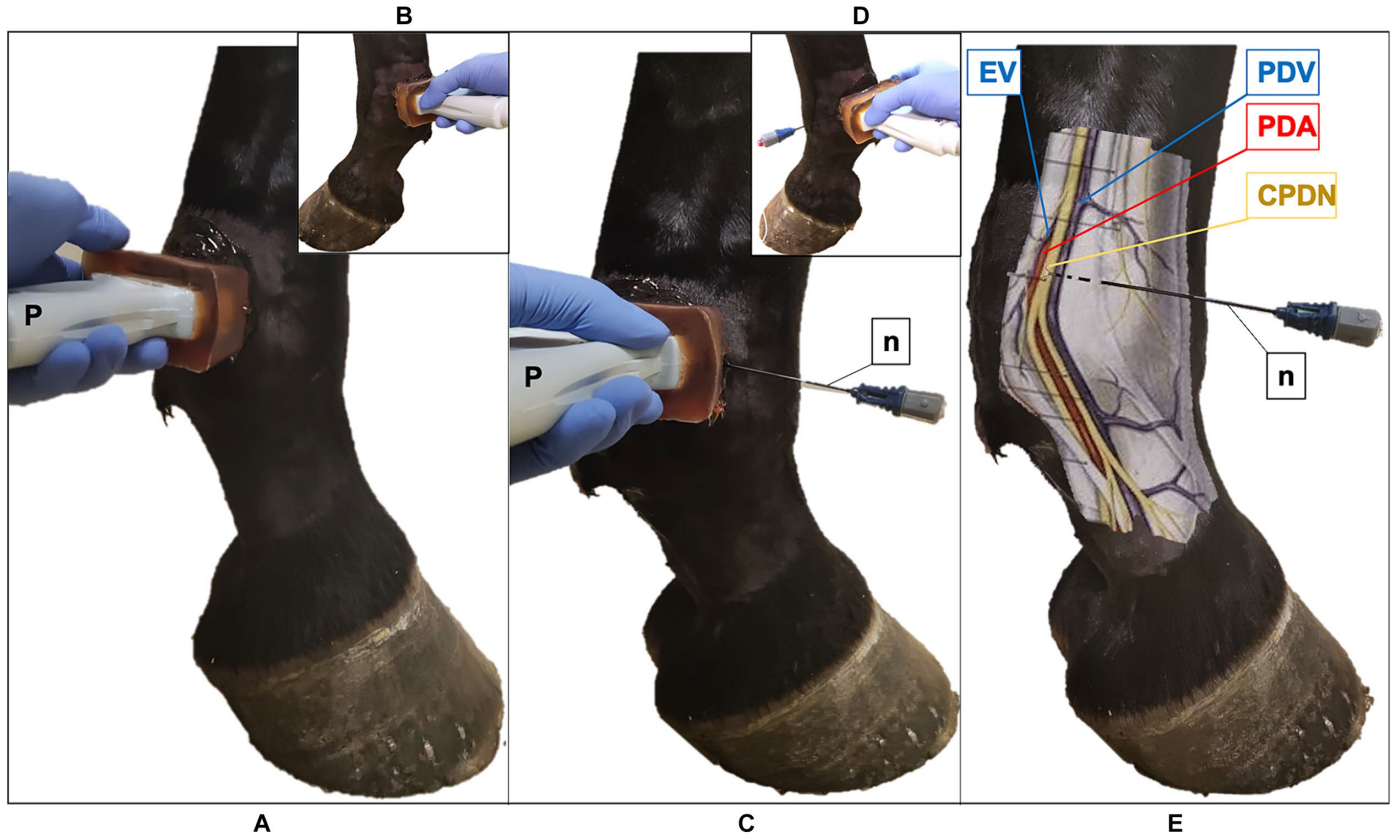
Ultrasound (US) probe and the radiofrequency (RF) needle positioning in the fetlock region of the equine forelimb **(A)** The US probe (P) is positioned transversal to the metacarpophalangeal joint at the level of the proximal portion of the proximal sesamoid bone, with the marker pointing dorsally; palmaro-medial approach **(B)** Palmaro-lateral approach. **(C)** US-guided in-plane technique, palmaro-medial approach: the RF needle (n) is inserted with a dorso-palmar direction to reach the common palmar digital nerve (CPDN). **(D)** Palmaro-lateral approach. **(E)** Anatomical representation of the medial neurovascular bundle: RF needle reaches the CPDN in the subcutaneous tissue. Note the presence of the ergot vein (EV) in the palmaro-medial approach, that is absent in the palmaro-lateral approach. PDV, palmar digital vein; PDA, palmar digital artery.

Ten horses referred to the Veterinary Teaching Hospital of the University of Milan undergoing diagnostic or surgical standing procedures not related to the current study were enrolled in phase I. Horses weighing less than 200 kg, less than 2 years of age or with current or previous episodes of forelimb lameness were excluded from the study. All other horses were considered eligible. Horses were restrained with the halter in a horse stock, in a quiet and clean area, in quadrupedal standing on a flat surface. The skin of the pastern and fetlock of both forelimbs was shaved, washed, and rinsed with alcohol. An acoustic standoff (Standoff pad; Esaote; Genova; Italy) was placed between the skin and probe to enhance visualization of superficial structures. To improve further acoustic coupling, a large amount of ultrasound gel (Ultrasound Gel; GIMA S.p.A.; Gessate; Italy) was applied to the probe. Ultrasonographic images were obtained using a portable US system (Sonosite M-Turbo) mounting a high-frequency 6–13 MHz linear array transducer (HLF38x; Sonosite Inc., WA, United States). The scanning depth was set at 2.2–2.7 cm and frequency was adjusted to obtain the clearer images as possible with less artifacts for each subject. An anesthetist experienced in US-guided loco-regional anesthesia collected the US images from both forelimbs. To identify the lateral and the medial PDNs in the pastern region, the US probe was positioned transversal to the middle third of the proximal phalanx, with the marker pointing dorsally and using a palmaro-lateral and a palmaro-medial approach, respectively (Figures 1A,B). Subsequently, the fetlock region was scanned with the same purpose; the US probe was applied transversal to the metacarpophalangeal joint with the marker pointing dorsally, and the US images were acquired using a palmaro-medial and palmaro-lateral approach, at the level of the proximal portion of the medial and lateral proximal sesamoid bone, respectively (Figures 2A,B). In the four examined regions, the anatomical structures and their echogenicity were identified, and a color flow Doppler was applied for a better identification of blood vessels and their relationship with adjacent anatomical structures. The US landmarks, target points and the best access point for an in-plane needle visualization were determined; the four sites studied in this phase were selected for RFA treatment in the next phases.

### 2.2 Phase II. US-guided RFA treatment and assessment of RF needle tip-to-nerve proximity: *ex vivo* study

**Figure 3:**
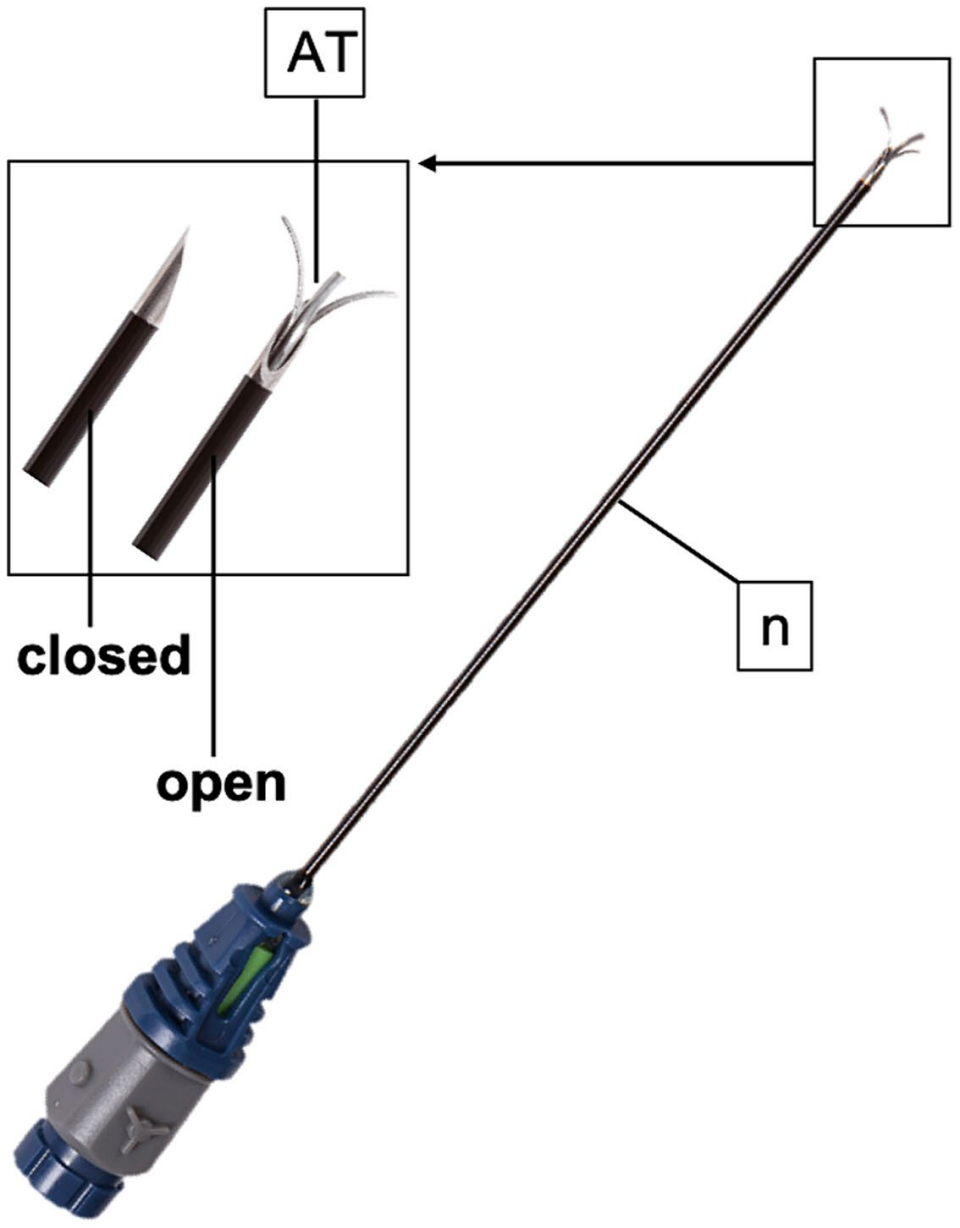
TRIDENT radiofrequency needle (n) used in the present study. The active tip (AT) possesses three tines that deploy at 10, 2 and 6 o’clock.

Phase II was performed on 10 fresh cadaver forelimbs: five right and five left forelimbs. Forelimbs were obtained from five horses euthanized for reasons not related to the present study. Limbs from horses weighing less 200 kg, less than 2 years old, or with a recent medical history of forelimb disorders were excluded. All other horses undergoing euthanasia were deemed suitable as donor. All forelimbs were separated at the carpus immediately after death and treatments were performed within 1 h from collection in a temperature-controlled examination room (21 ± 2°C). Each forelimb was divided into two regions, the pastern and the fetlock, further divided into lateral and medial sites. The skin of the pastern and fetlock was shaved and cleaned, and a dispersive return path electrode (GD-pad Corded; Diros Technology Inc., Ontario, Canada) was applied on the dorsal aspect of the proximal metacarpus and was connected to a RF generator (OWL URF-3AP RF Generator; Diros Technology Inc., Ontario, Canada). With the US technique described in phase I, the four sites selected for RFA treatment were examined and US landmarks were identified. An 18-gauge, three-tined, 5-mm active tip, 100-mm RF (TRIDENT) needle (RF Trident^™^ Cannulae; Diros Technology Inc., Ontario, Canada; Figure 3) was inserted using an in-plane US technique (Figures 1C,D, 2C,D). For the lateral pastern and fetlock, the RF needle was introduced in a dorso-lateral to palmaro-medial direction, while for the medial pastern and fetlock, in a dorso-medial to palmaro-lateral direction. The RF needle was inserted with an approaching angle of 0 to 30° to the sagittal plane until the needle tip reached the target PDN, thus maintaining a 90° needle inclination relative to the nerve axis (Figures 1E, 2E). The corresponding images were recorded and stored. Hence, RF needle tines were deployed and the RFA treatment performed. Four RFA treatments were tested: 60°C for 6 min (Group LOW), 70°C for 4 min (Group MEDIUM), 90°C for 2 min (Group HIGH) and 80°C for 8 min (Group VERY HIGH). The RFA treatment to be applied to each site was randomly selected,^1^ in order to obtain five trials for each treatment group in the pastern region, and five trials in the fetlock region. Both the region and the side from which to start, as well as the sequence of sites to be treated were randomly selected. Then, 0.1 mL of methylene blue was injected through the injection port of the RF cannula under real-time US guidance (22). After completing the described procedures, an experienced anatomist performed the cadaveric dissection to identify anatomical structures and tissues stained with methylene blue and to evaluate its proximity to PDNs (tip-to-nerve blue distance). For this purpose, the distance from the center of the colored tissues, which was assumed to have been the position of the active tip, and the target nerve, as well as the length of the stained nerve and its thickness were measured with a ruler (22). Any staining of blood vessels and non-target structures was also recorded. Moreover, the distance between the tip of the needle and the nerve was retrospectively measured on the stored US images by an anesthetist experienced in US-guided loco-regional anesthesia with the dedicated US software (tip-to-nerve US distance). All the described measurements were performed by the two independent authors, blinded to each other results, who performed each measurement once. The RF treatment was considered to have been carried out “at target” if at least 2 of the 3 following criteria were achieved: tip-to-nerve US distance less than 2 mm; tip-to-nerve blue distance less than 2 mm; length of stained nerve greater than 5 mm.

### 2.3 Phase III. Histological evaluation of nerve lesions induced by four RFA treatment protocols

For microscopical evaluation, all blue and grossly coagulated tissues, and a minimum of 2 cm^3^ of tissue including the treated PDNs and the corresponding artery and vein, were removed, laid onto a rigid surface and fixed with pins to avoid nerve contraction artifacts, and fixed in a 1/10 volume of 10% neutral buffered formalin for 48 h. Tissues were trimmed longitudinally and transversally and were routinely processed for 12 h, embedded in paraffin, cut in 5 μm sections and routinely stained with hematoxylin and eosin to assess thermal damage to the nerve and surrounding tissues. Negative controls consisted in nerves and adjacent tissues from the same forelimbs, collected 5 cm proximally from the RFA site. Negative controls were fixed and processed together with the coagulated nerves and were utilized for comparison. Histopathological alterations were evaluated twice by a ECVP diplomate veterinary pathologist who was blinded to the treatment group. The efficacy of RFA was evaluated microscopically by qualitative assessment of presence or absence of tissue coagulation and degeneration. Presence of axonal swelling and degeneration, collagen homogenation, nuclear smearing, or basophilia/amphophilia were considered as evidence of thermal injury, i.e., nerve coagulation when determined in live animals, on the basis of previously described morphologic features (12, 23). If partial coagulation of the nerve was observed, i.e., coagulation affecting part of the nerve but not its entirety, the case was considered as successful coagulation for statistical purposes.

### 2.4 Statistical analysis

Dedicated software for statistical analysis was used to perform all the evaluation described hereafter (JMP Pro, v. 17.0, SAS Institute, Cary, NC, United States; MedCalc version 19.2.6, MedCalc Software Ltd., Acacialaan 228,400 Ostend, Belgium). Data were tested for normality with the Shapiro–Wilk’s *W* test. Data were reported as mean ± standard deviation, median (range) or as number of samples (% of the total), where appropriate. To assess the statistical power of the study, we performed a *post hoc* power analysis for the chi-squared test using the contingency table which describe the distribution of nerve coagulation outcomes within each treatment group. With our observed effect size (1.70), an alpha error of 0.05% and 3 degrees of freedom, the analysis yielded a power estimate of 99%. The nerve thickness was compared between the four RFA treatment groups with the Kruskal Wallis test. The number of treatments “at target” was compared between the four RFA treatment groups with a contingency table and chi-square test. The association between nerve coagulation and axonal degeneration, nuclear smearing, and the coagulation of other structures (i.e., artery and collagen) was explored with a contingency table, and the chi-square or Fisher’s *F* test. A nominal logistic univariate analysis was performed to evaluate which factor influenced the presence of nerve coagulation at the histological evaluation, as the dependent variable. Moreover, the presence of other stained structures and the presence of other coagulated structures were separately evaluated as dependent variables. The following variables were evaluated as independent: treatment group (group LOW; group MEDIUM; group HIGH; group VERY HIGH), treated region (pastern; fetlock), nerve thickness (mm), tip-to-nerve US distance (mm), tip-to-nerve blue distance (mm), length of stained nerve (mm), target (yes; no), presence of other stained structures (artery; vein). *Post hoc* analysis was applied between significantly associated variables with the chi-square test for categorical variables and with the Mann–Whitney’s *U* test. Moreover, the association between the RFA treatment “at target” and the treatment region was evaluated with contingency table, and the chi-square or Fisher’s *F* test, as well. Nerve thickness was compared between the treatment regions with the Mann–Whitney’s *U* test. A Spearman’s rank correlation (*r_s_*) test was used to evaluate the strength of association between the tip-to-nerve US and blue distance measurements, and between these distances and the length of stained nerve. The overall ability of the US guided technique was evaluated by calculating the accuracy, sensitivity, specificity, positive and negative predicting values by comparing being “at target” or not with the presence/absence of nerve coagulation. The 95% confidence intervals (CI) were calculated as well. The analysis was repeated within each treatment group.

## 3 Results

**Figure 4:**
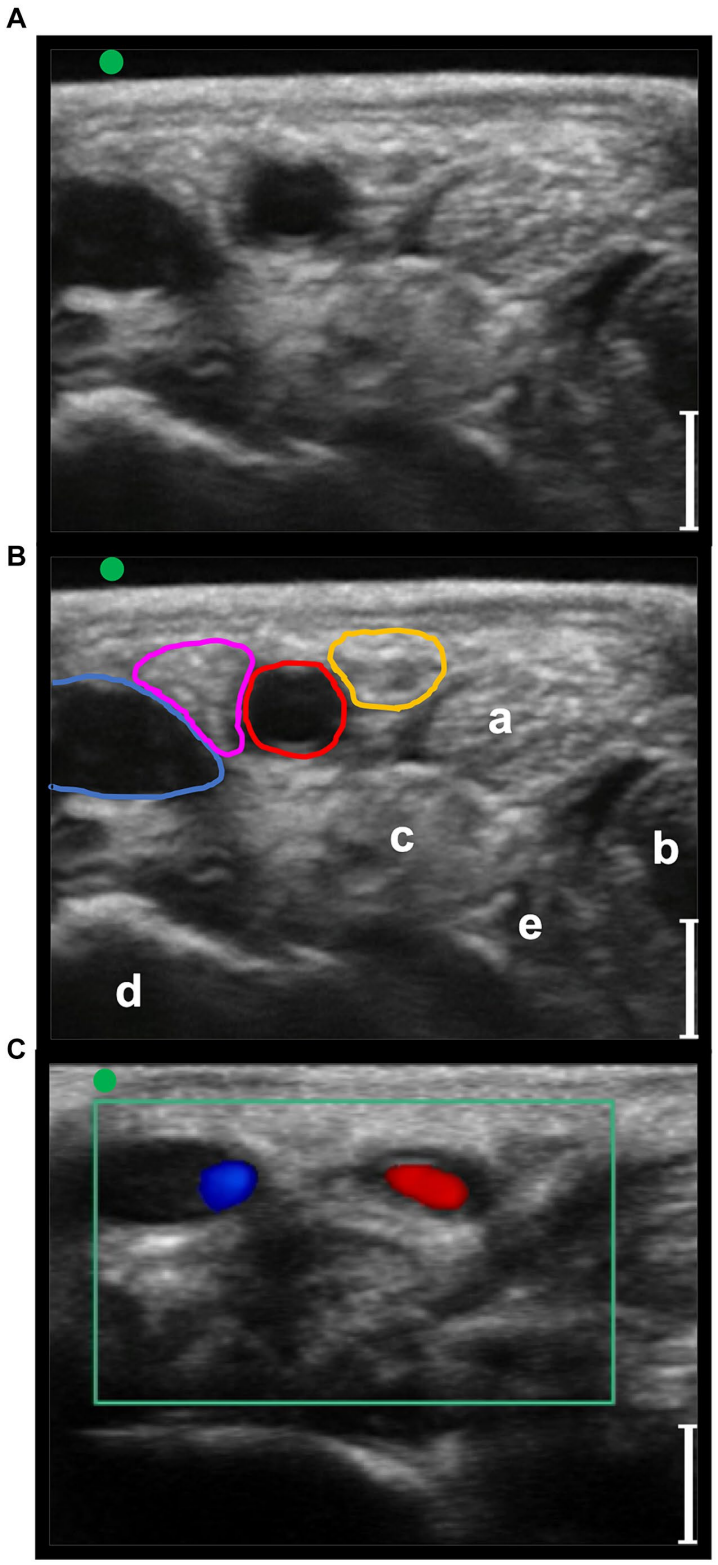
Transverse palmaro-lateral ultrasonographic images of the equine pastern at the level of the middle third of the proximal phalanx with the marker (green dot) pointing dorsally, *in-vivo*. **(A,B)** *in-vivo* scan. **(C)** *in-vivo* scan with color flow doppler. Blue line, lateral palmar digital vein; Red line, lateral palmar digital artery; yellow line, lateral proper palmar digital nerve; purple line, lateral dorsal palmar digital nerve branch of the middle phalanx; a, distal branch of the superficial digital flexor tendon; b, deep digital flexor tendon; c, oblique sesamoidean ligament; d, proximal phalanx; e, straight sesamoidean ligament; bars equal to 5 mm.

**Figure 5:**
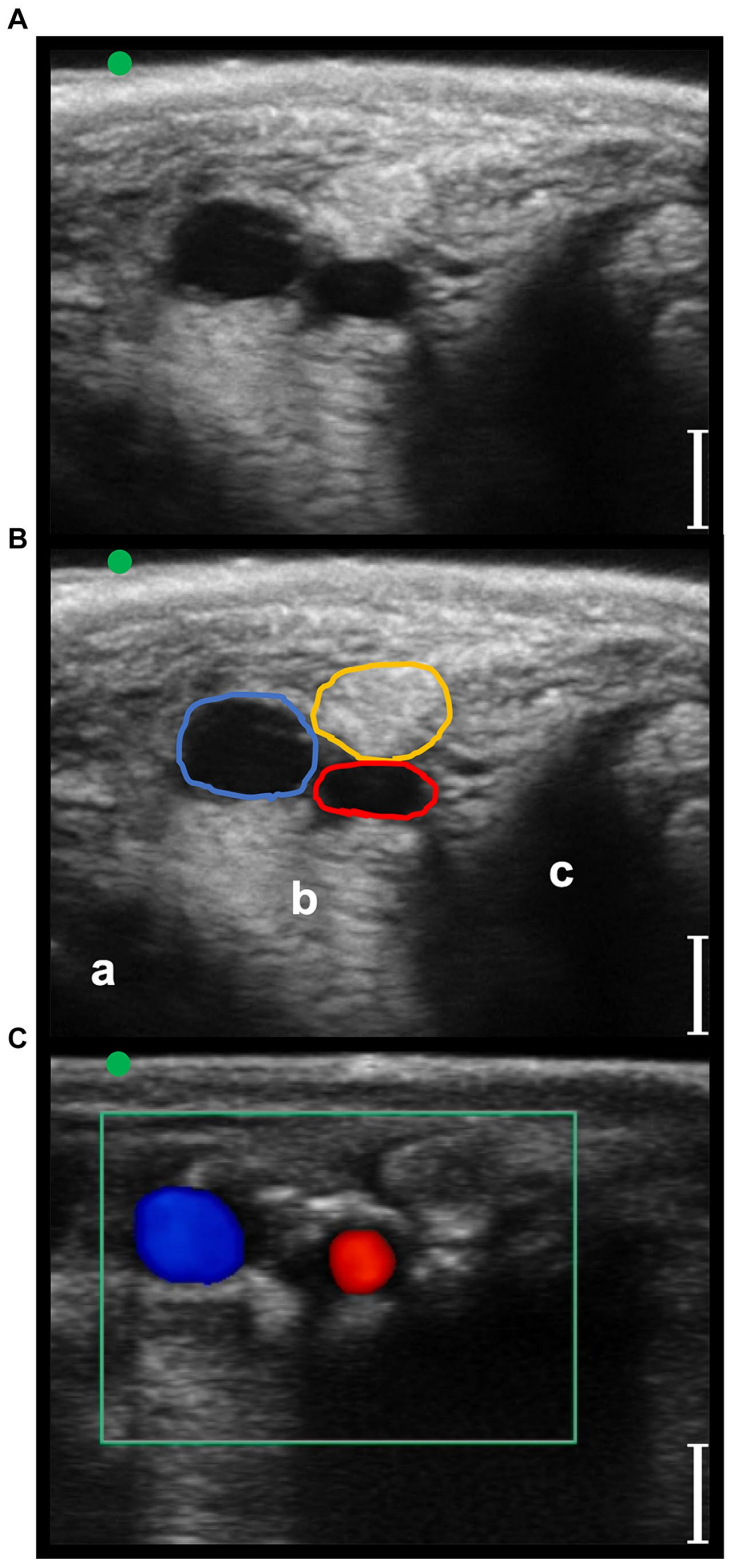
Transverse palmaro-lateral ultrasonographic images of the equine fetlock at the level of the proximal portion of the lateral proximal sesamoid bone, with the marker (green dot) pointing dorsally, *in-vivo*. **(A,B)** *in-vivo* scan. **(C)** *in-vivo* scan with color flow doppler. Blue line, lateral palmar digital vein; Red line, lateral palmar digital artery; yellow line, lateral common palmar digital nerve; a, third metacarpal bone; b, lateral branch of the suspensory ligament; c, lateral proximal sesamoid bone; bars equal to 5 mm.

In the phase I, a total of 20 forelimbs from 10 horses were examined, obtaining the evaluation of 20 lateral and 20 medial pasterns, and 20 lateral and 20 medial fetlock regions. The position of the different anatomical structures and their relationship with the neurovascular bundles were considered consistent in all the limbs evaluated. Transverse palmaro-lateral and palmaro-medial US images of pastern (Figure 4) and fetlock (Figure 5) regions allowed easy identification of the neurovascular bundle close to the skin surface in all horses. In the pastern region, the neurovascular bundles were superficial to the distal branch of the superficial digital flexor tendon and the deep digital flexor tendon. More deeply, the proximal phalanx, the oblique and the straight sesamoidean ligament were visualized (24). In the fetlock region, the neurovascular bundles were superficial compared to the third metacarpal bone, the lateral or medial branch of the suspensory ligament and the corresponding proximal sesamoid bone (25). In both regions, the palmar digital veins (PDVs) showed an anechoic vascular bed with a thin wall easily compressible, whereas the palmar digital arteries (PDAs) were smaller, anechoic, round, pulsatile structures with a thicker wall. In addition, in the transverse palmaro-medial scan of the fetlock, the ergot vein, after branching from the medial PDV, was always visible, palmar to the medial PDN. The PDN was always observed palmar to the PDV both on the medial and lateral sides, with a coarse grainy appearance and a clear distinction from the subcutaneous tissue. In the pastern region, the proper PDNs and their intermediate *rami*, i.e., the dorsal PDN branch of the middle phalanx, were always identified palmar and dorsal to the PDA, respectively (26). In the fetlock region, the lateral and medial common PDNs were superficial to the lateral and medial PDAs, before branching into the lateral and medial proper PDNs and their corresponding dorsal *rami*, i.e., dorsal PDN branch of the proximal phalanx, which were observed running palmar and dorsal to the PDA, respectively (26).

**Figure 6:**
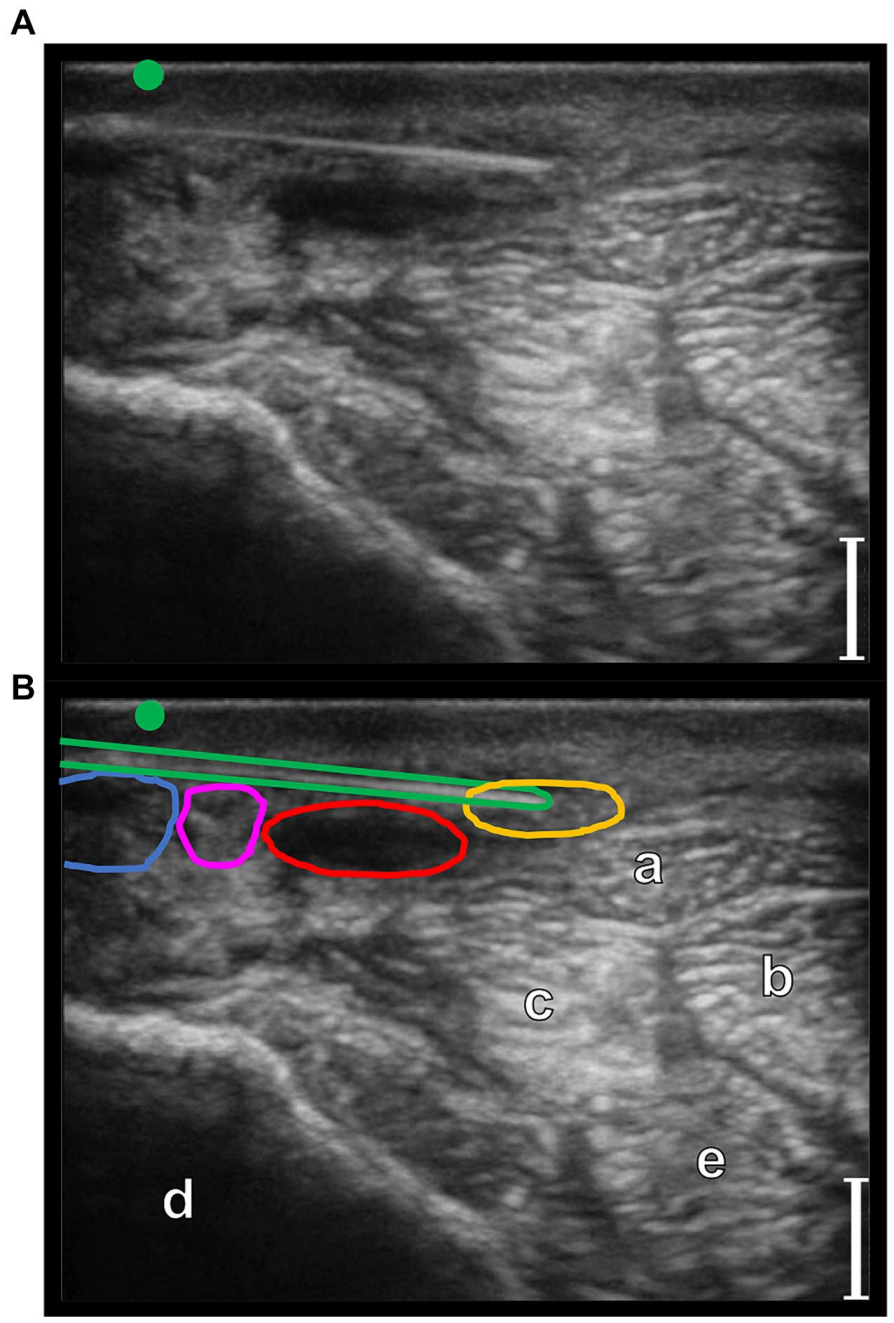
Transverse palmaro-lateral ultrasonographic image of the equine pastern at the level of the middle third of the proximal phalanx with the marker (green dot) pointing dorsally, *ex-vivo*. **(A,B)** *ex-vivo* scan. Radiofrequency needle (green line) positioning on the target before opening the tines. Blue line, lateral palmar digital vein; Red line, lateral palmar digital artery; yellow line, lateral proper palmar digital nerve; purple line, lateral dorsal palmar digital nerve branch of the middle phalanx; a, distal branch of the superficial digital flexor tendon; b, deep digital flexor tendon; c, oblique sesamoidean ligament; d, proximal phalanx; e, straight sesamoidean ligament; bars equal to 5 mm.

**Figure 7:**
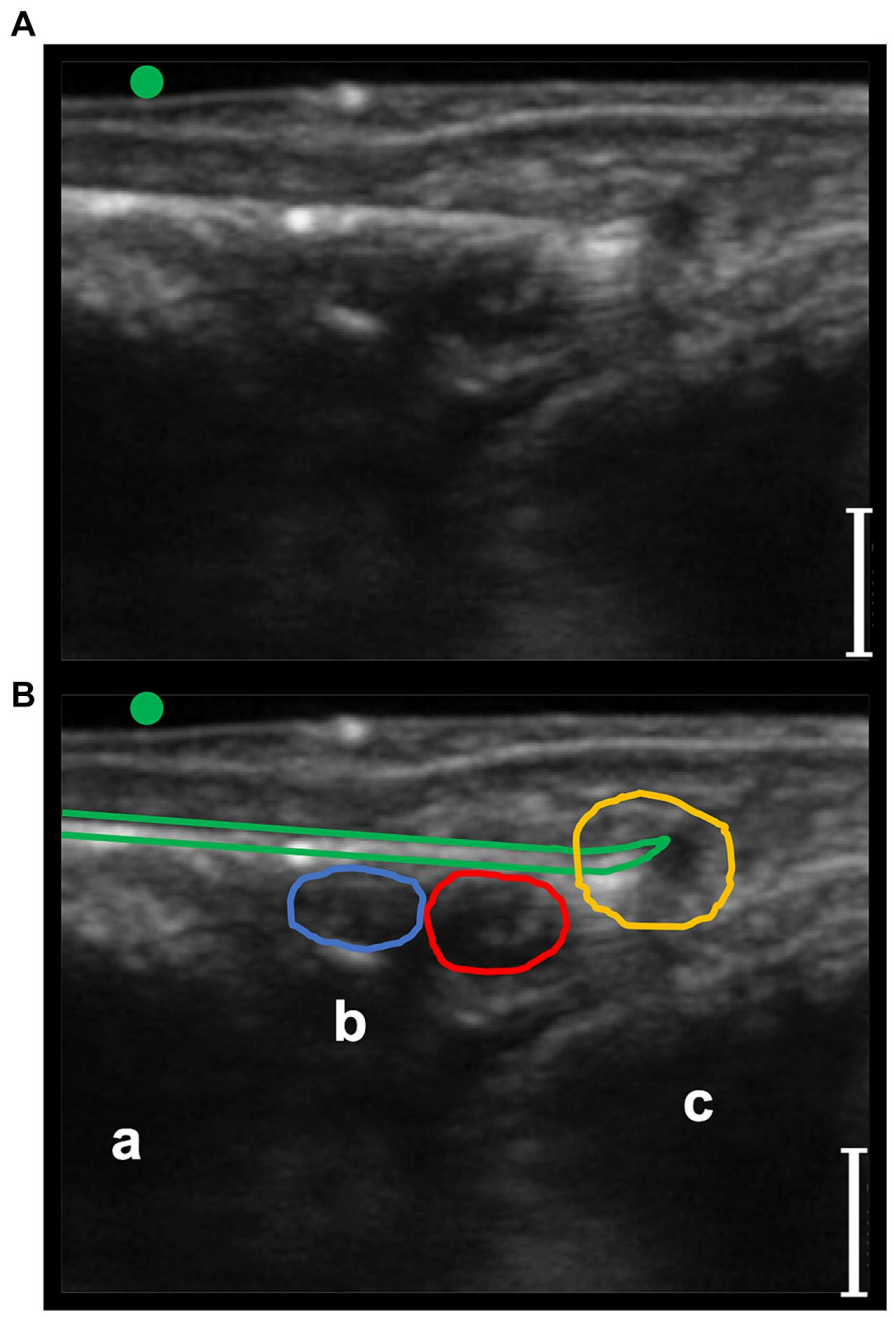
Transverse palmaro-lateral ultrasonographic image of the equine fetlock at the level of the proximal portion of the lateral proximal sesamoid bone, with the marker (green dot) pointing dorsally, *ex-vivo.* **(A,B)** *ex-vivo* scan. Radiofrequency needle (green line) positioning on the target after opening the tines in the cadaver forelimb. Blue line, lateral palmar digital vein; Red line, lateral palmar digital artery; yellow line, lateral common palmar digital nerve; a, third metacarpal bone; b, lateral branch of the suspensory ligament; c, lateral proximal sesamoid bone; bars equal to 5 mm.

In phase II and III, a total of 10 forelimbs, resulting in 10 lateral and 10 medial fetlocks and 10 lateral and 10 medial pasterns, were included. All the anatomic structures of interest studied during the *in vivo* phase I were detectable in the phase II *ex vivo* experiment. The RF needle was clearly ultrasonographically identifiable, as well (Figures 6, 7).

No significant differences were observed between the four RFA treatment groups regarding nerve thickness and number of treatments performed “at target.” Results are summarized in Table 1. The number of treatments “at target” were 31 (77.5%); of which four did not display all the three criteria to be categorized as “at target.” In particular, three had a higher tip-to-nerve blue distance, and one displayed a shorter length of stained nerve.

**Table 1:** Mean ± standard deviation or median (range) of the nerve thickness (in mm) and proportion of radiofrequency ablation (RFA) treatments “at target” in the four groups (*n* = 10 per group).

Treatment group	Nerve thickness (mm)	RFA “at target”
Low	4.5 (3.0–8.0)	8/10
Medium	4.8 ± 1.1	8/10
High	4.9 ± 1.8	7/10
Very High	5.0 (3.0–9.0)	8/10
	Kruskal-Wallis *p* = 0.88	Chi-square *p* = 0.93

The *p* values represented the difference between the four treatment groups.

**Figure 8:**
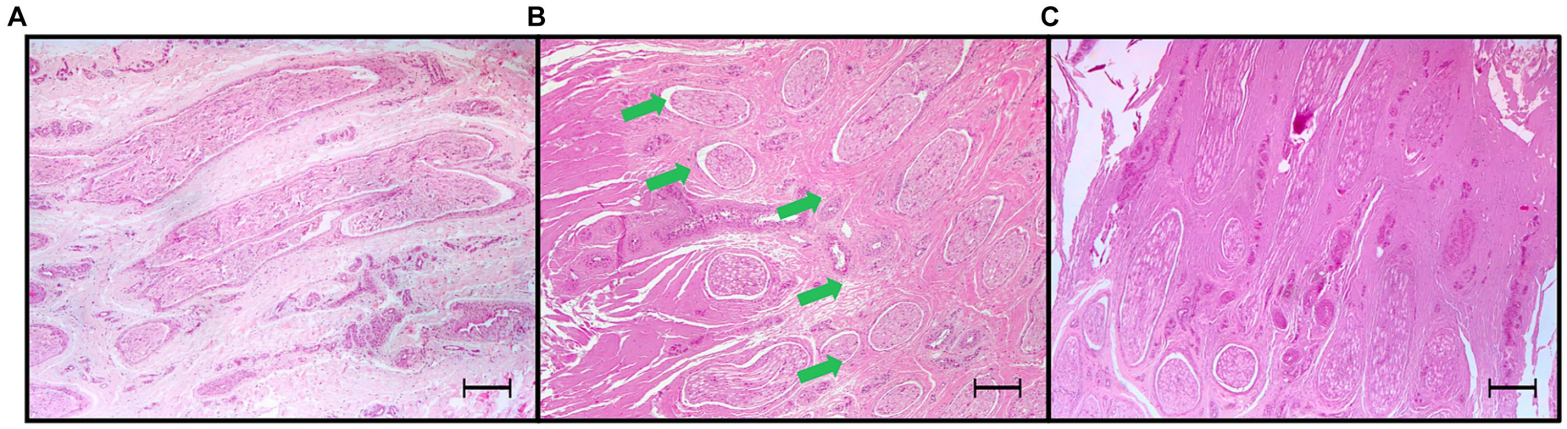
Light microscopic images (4x) of hematoxylin and eosin-stained sections of palmar digital nerves harvested from equine cadaveric forelimbs after radiofrequency ablation treatment. **(A)** Negative control: normal microscopical features. **(B)** Radiofrequency ablation treatment (Group MEDIUM: 70°C, 4 min): partial coagulation of the nerve. The radiofrequency treatment caused coagulation of tissues characterized by homogenation, loss of fibrillary patterns, increased eosinophilia and basophilia, leaving a portion of the palmar digital nerve undamaged (green arrows). **(C)** Total coagulation of the nerve tissue (Group VERY HIGH: 80°C, 8 min). Note the increased basophilia/amphohilia of the collagen in correspondence of the coagulated areas. Bars equal to 250 μm.

**Figure 9:**
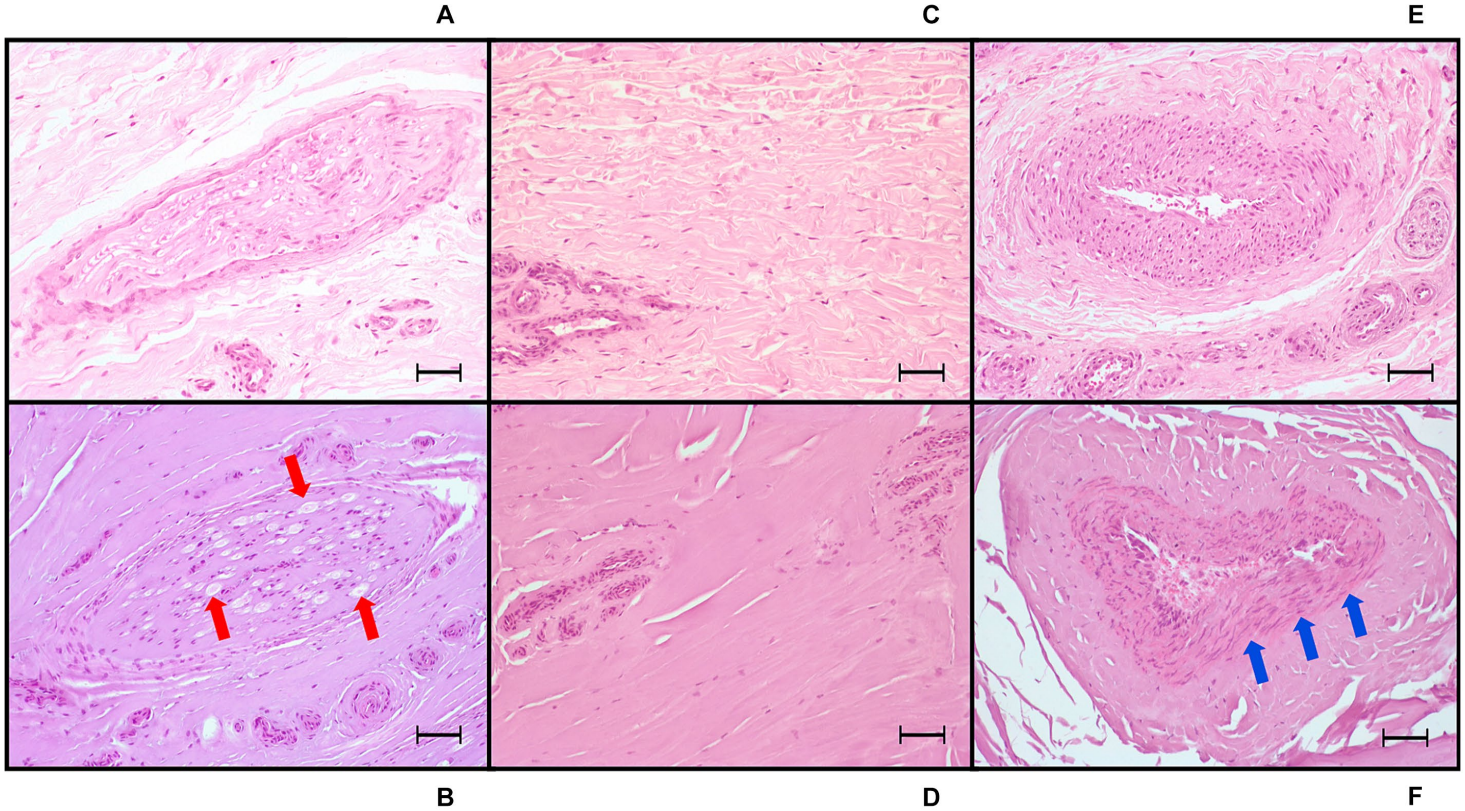
Light microscopic images (20x) of hematoxylin and eosin-stained sections of tissue treated with radiofrequency ablation, harvested from equine cadaver forelimbs. **(A,C,E)** Negative controls of nerve, collagen, and arterial tissue, respectively. **(B)** Radiofrequency ablation treatment (Group VERY HIGH: 80°C, 8 min): nerve coagulation, with axonal swelling and degeneration, increased intracytoplasmic vacuoles (red arrows) and cytoplasmic eosinophilia, perineurial sheath homogenation (coagulation) and swelling. **(D)** Extensive collagen coagulation with homogenation and loss of the fibrillar pattern. **(F)** Arterial wall coagulation: presence of severe nuclear smearing (blue arrows). Bars equal to 50 μm.

In the four treatment groups, histological examination of the PDNs was consistent with thermal injury. Lesion comprised oedema, increased intracytoplasmic clear empty axonal vacuoles (hydropic degeneration), degeneration of nerve sheaths and hypereosinophilia of axons. Also, collagen homogenation that appeared intensely eosinophilic with loss of distinct borders, loss of fibrillar pattern interpreted as coagulation (27), and areas of increased basophilia/amphophilia ascribe to electrical impulse tissue damage (Figures 8, 9). In the arterial walls of the coagulated areas variably severe nuclear changes including pyknotic, fusiform nuclei (nuclear smearing) were consistently present (Figure 9F). Lesions were readily visible in longitudinal sections comprising the entire length of the nerves examined, while transverse sections often did not include the specific area of nerve/tissue damage.

Presence of nerve coagulation was significantly associated with axonal degeneration, nuclear smearing and presence of other coagulated structures. The specific results are reported in Table 2. No association was observed between coagulated non-target structures and treatment groups (*p* = 0.32), even when considering if the RF was performed “at target” (*p* = 0.16) or not (*p* = 0.53).

**Table 2:** Number of axonal degeneration, nuclear smearing, and coagulation of other structures in the presence or absence of nerve coagulation.

		Nerve coagulation		
		Yes	No	
Axonal degeneration	Yes	18	0	Fisher’s F *p* < 0.001
	No	0	22	
Nuclear smearing	Yes	17	10	Fisher’s F *p* = 0.002
	No	1	12	
Other coagulated structures	Artery	0	3	Chi-square *p* < 0.001
	Artery and collagen	17	7	
	Collagen	1	0	
	No	0	12	

These tests were performed on the overall sample (*n* = 40).

Treatment group significantly influenced the presence of nerve coagulation, with the VERY HIGH group showing the highest and the LOW group the lowest frequency of coagulated nerves, even when only treatments performed “at target” were considered. In contrast, when RF did not result “at target,” no nerve coagulation was observed in any group. The specific results are reported in Table 3.

**Table 3:** Number of presence/absence of nerve coagulation in radiofrequency ablation (RFA) treatment groups and considering only RFA treatments performed “at target” (*n* = 10 per group).

Treatment group	Nerve coagulation		Nerve coagulation in RFA “at target”	
	Yes	No	Yes	No
Low	1	9	1	7
Medium	4	6	4	4
High	5	5	5	2
Very High	8	2	8	0
	Chi-square *p* = 0.03		Chi-square *p* < 0.001	

The *p* values represented the difference between the four treatment groups.

The treated site did not show any influence on the presence of nerve coagulation (*p* = 0.53); nonetheless, the treated site was significantly associated with the number of treatments “at target,” with a higher number of failures in the fetlock region (Table 4).

**Table 4:** Number of radiofrequency ablation (RFA) treatments performed “at target” in fetlock and pastern region (*n* = 20 per region).

	RFA treatment “at target”	
	Yes	No
Fetlock	12	8
Pastern	19	1
	Fisher’s F *p* = 0.008	

The nerve thickness did not show any influence on the presence of nerve coagulation (*p* = 0.25); nevertheless, the nerve thickness resulted significantly higher (*p* < 0.0001) in the fetlock region (6; 4–9 mm) compared to the pastern region (4; 3–6 mm).

The tip-to-nerve US and blue distances and the length of stained nerve resulted significantly associated with the presence of nerve coagulation, and all resulted significantly correlated to each other. The tip-to-nerve US and blue distances resulted associated with staining and coagulation of non-target structures. No association was observed between stained and coagulated non-target structures (*p* = 0.36). The specific results are summarized in the Supplementary Tables 1, 2.

Overall, the US-guided technique showed an accuracy of 67.5%, with a sensitivity of 100% and specificity of 41%. The specific results in the whole sample and within each treatment group are reported in Table 5.

**Table 5:** Sensitivity, specificity, PPV and NPV of the US-guided technique compared to the histologic confirmation of nerve coagulation (overall = 40; *n* = 10 per group).

Treatment group	Sensitivity (%)	Specificity (%)	PPV (%)	NPV (%)
Overall	100 (81.5–100)	41 (21–64)	58 (49–66)	100 (66–100)
Low	100 (2.5–100)	22 (3–60)	12.5 (9–17)	100 (16–100)
Medium	100 (40–100)	33 (4–78)	50 (36–64)	100 (16–100)
High	100 (48–100)	60 (15–95)	71 (46–88)	100 (29–100)
Very High	100 (63–100)	100 (16–100)	100 (63–100)	100 (16–100)

PPV, positive predictive values; NPV, negative predictive values; US, ultrasound.

## 4 Discussion

In human medicine, RF has been employed for many years, and numerous studies have reported its use to treat hand and foot neuropathic pain (28–30). To the authors’ knowledge, only one preclinical experimental study investigating histological and electrophysiological effects of RF technique on the canine sciatic and saphenous nerves is currently available (18), whereas no reports are available for horses. Distal forelimbs are one of the most common sites of lameness in horses (31–33). Since chronic lameness is often difficult to treat, manage, and resolve (4), and represents a major cause of reduced quality of life in horses (1), additional options to control lameness induced by chronic pain are desirable. Hence, this study was designed to identify US anatomical landmarks in horses with the perspective of applying this technique in clinical cases.

Ultrasonography was used to identify the optimal sites of treatment for its confirmed ability to easily identify anatomical landmarks, since it provides optimal visualization of soft tissues compared to other techniques, such as fluoroscopy (23). For this reason, the US technique ensures accurate positioning of the needle close to the target and increases the procedure’s safety (34). The equine pastern and fetlock regions have been studied with US (24, 25), but without focusing on the identification of a specific US window for the recognition of the PDNs. Indeed, in clinical practice, diagnostic analgesia is performed using a blind technique, and the nerve is identified only by palpation (35, 36). In the present study, transverse palmaro-lateral and palmaro-medial US images allowed for straightforward detection of the neurovascular bundle close to the skin surface in all horses. In addition, the use of color flow Doppler further facilitated the identification of blood vessels and their distinction from the nerve *in vivo*. The transverse section of the PDN was easily distinguishable from the subcutaneous tissue and it was always visualized adjacent to the PDA, confirming the first hypothesis of this study. Hence, according to the primary aim, the PDA should be considered the best US landmark for the identification of PDNs in both the pastern and fetlock regions.

The second aim of this study was to evaluate the feasibility of an US-guided RFA treatment in horses using an *ex vivo* model. Due to the nerve proximity to critical anatomical structures and the requirement for precise targeting with a RF needle, phase II of the study employed the in-plane US technique for its intuitiveness and safety (34, 37). The RF needle was carefully advanced through the subcutaneous tissue in a dorso-lateral to palmaro-medial direction for lateral fetlock and pastern and in a dorso-medial to palmaro-lateral direction for medial fetlock and pastern and with an approaching angle of 0 to 30° to the sagittal plane until it reached the nerve, thus maintaining a 90° needle inclination relative to the nerve axis. Finlanson and colleagues (2017) proved that the standard RF needle, whose lesion is ellipsoidal around the active tip, requires a parallel approach to the target and loses effectiveness as the angle of inclination to the nerve increases (38). In contrast, the caudal deployment mechanism of the three tines of the TRIDENT needle which deploy at 10, 2 and 6 o’clock (38) allows the formation of a 3-dimensional “pear-shaped” lesion, thus increasing the distal width of the lesion, but varying minimally its length (39). This pyramid-like conformation is less sensitive to the actual inclination of the needle with respect to the nerve, with similar morphologies at angles of 0 and 90 degrees, and the development of a larger lesion surface area at 90 degrees compared with other RF needles (38). For these reasons, this particular RF needle was chosen for the in-plane US approach applied in the present study. Moreover, the tines are flexible and, once the active needle tip is in contact with the target nerve, they are deployed and advanced 2 mm to easily surround the nerve, further increasing the surface area involved by the RFA. Based on manufacturer unpublished data, in a standard model, the estimated lesion size generated by RFA with the TRIDENT needle ranges from 0.5×0.5 mm to 10×10 mm, based on the temperature/min settings of the RF generator. The US technique applied in the present study enabled the needle to be correctly positioned on the target nerves with a success rate of 77.5%. The altered echogenicity of the tissues, the absence of blood flow through the vessels, and the impossibility of using color flow Doppler, made accurate identification of the anatomical landmarks and the nerve more difficult in the phase II *ex vivo* study, likely further increasing the failure rate (40). The higher incidence of failures observed in the fetlock region is possibly attributable to a larger amount of subcutaneous tissue compared with the pastern region, which could have increased nerve mobility allowing for its caudal shifting upon needle insertion. Furthermore, the proximal sesamoid bone curved surface likely exacerbated challenges in maintaining optimal contact between the probe and the skin, thus increasing the difficulty of accurately positioning the needle close to the nerve. Considering the purpose of the present study, the positioning of the needle tip had to be assessed very precisely. To this end, the volume of methylene blue injected was very small, according to a previous human cadaveric study (22). In contrast, previous studies evaluating the accuracy of US-guided peripheral nerve blocks in equine cadavers used considerably larger volumes of dye (41). However, the dispersion of dye within cadavers may not accurately reflect the spread of the injectate in live animals (42). Indeed, methylene blue evaluation is limited by the bias of color spread in the tissues, and by its potential drainage in lymphatic vessels (43), and it cannot be evaluated *in vivo*. For this reason, the needle tip-to-nerve US distance was also assessed. Nonetheless, the tip-to-nerve blue distance also proved to be reliable in identifying correct needle positioning and strongly correlated with the US distance. Indeed, it was observed that both US and blue needle tip-to-nerve distances were significantly associated with the presence of coagulation, highlighting the importance of an extremely precise RFA needle positioning. Thus, despite the use of strict evaluation criteria that integrate two different methodologies to delineate the precise positioning of the RF needle, i.e., methylene blue and US, and the procedure was deemed successful when two out of the three proposed criteria were fulfilled, the US-guided RFA treatment has yet demonstrated a high success rate.

The third aim of this study was to assess the microscopical changes produced on PDNs, which are responsible for the transmission of pain signal from the distal limb. In the present study, histopathological lesions observed in PDNs were always consistent with axonal thermal damage, aligning with the existing literature (12, 23, 44). The application of RFA heat above 60°C led to rapid protein denaturation and extensive tissue coagulation (45, 46). According to Seddon’s classification (47), axonotmesis occurred, with disruption of axons, myelin, and supporting connective tissue except for the epineurium (17). Following Sunderland’s more detailed classification (48), a third-or fourth-degree peripheral nerve injury was generated, thus involving also the endoneurium or even the perineurium, respectively (46). When considering the *in vivo* effects, the thermal neurolysis produced by RFA has been reported to produce shortly thereafter Wallerian degeneration, initially developing at the target site, microscopically characterized by axonal swelling, fragmentation, and secondary demyelination (18); thereafter, the remaining distal portions of the axon and its myelin sheath also degenerate (46). This process causes transitory interference with nerve transmission, resulting in temporary denervation and pain relief (17). With an increasing degree of severity of axonal injury, the ability and rate of axonal regeneration diminishes with a longer duration of pain relief that can last months to years (46), but with a potential for neuroma-in-continuity formation in fourth-degree injuries (49). Indeed, in humans, post-procedural complications are considered extremely rare, and the most reported is neuropathic pain (50). Unfortunately, due to the *ex vivo* nature of this study, the clinical grade of the peripheral nerve lesions and their progression could not be evaluated as it was not possible to assess either the duration of pain relief or the possible occurrence of post-treatment complications. Currently, unresponsive chronic pain in the horse is finally dealt with surgical neurectomy, which has been associated with an increased risk of distal limb injuries, such as lesions at the deep digital flexor tendon, luxation of the distal interphalangeal joint, and sub-solar injuries (9). It cannot be anticipated if these complications may arise also after the RFA treatment. Future clinical studies are needed to elucidate these aspects. Finally, we could confirm that increasing the RFA treatment intensity determined a higher PDNs coagulation effectiveness. Indeed, beyond the already discussed needle inclination, size, active tip length, and the number of tines, it should be bore in mind that according to the literature, the size of lesions induced by RF is influenced by the temperature and the treatment duration (51). In the current study, four RFA treatments employing varying temperature/min settings underwent evaluation: the LOW and VERY HIGH treatments were selected to assess the effect of extreme settings. The MEDIUM treatment served as an intermediary setting positioned between the aforementioned groups, while the HIGH treatment applied settings often employed in human clinical studies (52, 53). Although it has been reported that larger lesions can improve treatment outcomes and increase the duration of pain relief in humans (54), the proximity of the nerve trunk to other important structures such as the arteries that need to be preserved (17) must be considered. Furthermore, in humans, nerve tissue is the least resistant to electric current passage and it is therefore the most vulnerable to electric-thermal damage (44). On the contrary, blood vessels would be less sensitive due to the blood flow dissipating hyperthermia, the heat sink effect (45). Therefore, it might be hypothesized that a precise, small, and localized RF-induced lesion would be sufficient to involve the nerves without damage to adjacent relevant anatomical structures. However, it has been reported that thermal injury caused by RFA might be too small, thus failing to induce complete denervation (17). Partial denervation may lead to diminished extent and duration of pain relief, potentially resulting in early recurrence of pain or lack of pain elimination (12). In the present study, the success rate of nerve coagulation was significantly higher as the RFA intensity settings increased, with the highest frequency of coagulated nerve in VERY HIGH group but did not differ between the treatment sites. Also, partial nerve coagulation was observed in LOW and MEDIUM groups. Specifically, within the LOW group, the single case displaying nerve lesions exhibited only partial coagulation. In addition, in the MEDIUM treatment group, 3 out of 4 coagulated nerves were characterized by partial coagulation at the histologic evaluation. Due to these findings, the effectiveness of these two treatments in providing pain relief *in vivo* is at least unlikely for the LOW treatment group and remains to be explored at the clinical level. Applying the LOW and MEDIUM treatments in the effort of minimizing damage to non-target tissue should be avoided, since no significant differences in coagulation of non-target structures were observed between treatment groups. This was likely due to the size of lesions that was always enough extensive to involve the PDA, since the artery is very close to the PDNs. In fact, the correct needle position and the finding of coagulation were associated with nuclear smearing of the arterial leyomyocytes. These microscopical findings are in agreement with previous descriptions (12, 23, 44). Similar lesions have been also reported in humans, but in contrast to the findings from this work, they have been more commonly associated with the application of high-intensity electrical or high-power laser devices (55). Regarding PDVs, as the distance increased, a significant increase in vein blue dye staining was observed, but the PDV was never coagulated. This result may likely derive from the altered dye spread in cadaveric tissues and the greater distance of the PDVs with the PDNs. These findings are important to consider for the *in vivo* application of this technique, as the use of RFA in the fetlock and pastern regions could result in the development of collateral damage on non-target anatomical structures, especially the PDA. Nonetheless, both clinical and experimental PDAs damage did not provoke clinically relevant consequences as collateral circulation developed rapidly and tissue necrosis did not develop (56–59). Noteworthy, it has been previously described that in cadaveric limbs the absence of vascular heat sink effect may be a possible cause of overestimation of the collateral thermal injury compared to the *in vivo* effects of RFA treatment mitigated by adequate blood flow (45). Hence, potential RFA induced damage of the PDA should be explored in future studies to assess its clinical relevance.

This study retains some limitations, in addition to those already mentioned, all possibly related to the use of cadaveric limbs. The US window assessment in phase I was performed on living horses in standing position, while RF needle placement in phase II was not performed with a weight-bearing limb. This may have altered the relationships between anatomical structures and affected the success rate of both the US technique and the RFA treatments. A cut-off value for the US distance between the nerve and the active needle tip, beyond which coagulation did not occur was not evaluated. It would be interesting to ascertain such a value to be applied in the clinical setting. Furthermore, the extension of the lesions generated by the RFA treatments may differ from those generated in live animals due to different electrothermal properties and temperature of the tissue. Similarly, histological findings might not be comparable to those *in vivo* because of the absence of both vascular and cellular reactions in dead tissues and of the thermal dissipating ability of the blood flow. Finally, further clinical research is deemed necessary to determine the efficacy and duration of pain relief, to assess the emergence of potential complications, and to allow the definition of the optimal procedural parameters *in vivo*.

## 5 Conclusion

In conclusion, with transverse palmaro-lateral and palmaro-medial US images of fetlock and pastern regions the palmar digital neurovascular bundles in horses were easily detected. *In vivo*, color flow Doppler imaging allowed a better distinction of blood vessels from nerves, and visualized the PDN consistently adjacent to the PDA, which was deemed as the best US landmark. Despite the need of extremely precise RF needle positioning, the use of the in-plane US technique for this purpose proved to be successful, with a 77.5% of success rate of placing the needle “at target,” albeit with some greater difficulty in the fetlock region. The use of a TRIDENT needle, which creates a “pear-shaped” lesion with a larger distal ablative area that is less sensitive to angulation changes, probably improved the efficacy of the procedure, despite a 90 degrees needle-to-nerve angle. Furthermore, the tip-to-nerve distance significantly affected the presence of nerve coagulation, highlighting the importance of positioning the RF needle as close to the target nerve as possible. In all groups, the histopathological findings revealed consistent axonal degeneration induced by RFA on PDNs of horses, aligning with the existing literature. The temperature/min setting in the treatment groups was significantly associated with the success rate of nerve coagulation, but not with coagulation of non-target structures. On the basis of the obtained results, HIGH and VERY HIGH RFA treatments might be worth of being applied in future *in vivo* clinical studies, focusing on treating chronic lameness of the distal forelimb in horses, since these protocols could potentially display an effective and long-lasting pain relief. Nonetheless, it is important to consider that potential complications, if any, might occur after the RFA treatment.

## Data availability statement

The original contributions presented in the study are included in the article/Supplementary material, further inquiries can be directed to the corresponding author.

## Ethics statement

The animal studies were approved by Institutional Ethical Committee for Animal Care at the University of Milan (OPBA) n° 51_2023. The studies were conducted in accordance with the local legislation and institutional requirements. Written informed consent was obtained from the owners for the participation of their animals in this study.

## Author contributions

MA: Conceptualization, Data curation, Investigation, Methodology, Project administration, Writing – original draft. VR: Data curation, Investigation, Methodology, Resources, Writing – original draft. GR: Conceptualization, Investigation, Methodology, Project administration, Supervision, Validation, Writing – review & editing. LA: Formal analysis, Visualization, Writing – review & editing. FB: Investigation, Validation, Writing – review & editing. PiR: Resources, Visualization, Writing – review & editing. SD'A: Data curation, Validation, Writing – review & editing. PaR: Conceptualization, Data curation, Formal analysis, Methodology, Resources, Supervision, Writing – review & editing.

## Footnotes

^1^ www.randomizer.org
