# Supplementary material for: Development of an ultrasound-guided radiofrequency ablation technique in the equine cadaveric distal limb: histological findings and potential for treating chronic lameness
Source: Front Vet Sci. 2024 Aug 23;11:1437989. doi: 10.3389/fvets.2024.1437989 (PMC11377333; doi:10.3389/fvets.2024.1437989)
Supplement: Supplementary file 1 [file Table_1.docx]

Supplementary Material

**Supplementary Table 1**. Association between the tip-to-nerve ultrasound (US) and blue distances and the length of the stained nerve (in mm) with the presence of nerve coagulation.

| **Measurement (mm)** | **Logistic estimates** | **Nerve coagulation** | | **Mann-Whitney’s U test** |
| --- | --- | --- | --- | --- |
|  |  | **yes** | **no** |  |
| **Tip-to-nerve US distance** | *p* = 0.009 | 0 (0 – 1.9) ^†^ | 0.8 (0 – 2.8) ^‡^ | *p* < 0.001 |
| **Tip-to-nerve BLUE distance** | *p* = 0.04 | 0 (0 – 5) ^†^ | 0 (0 – 20) ^‡^ | *p* = 0.01 |
| **Length of Stained Nerve** | *p* = 0.01 | 15.5 (8- 25) ^‡^ | 9.5 (0 – 25) ^†^ | *p* = 0.02 |

‡ > † for *p* < 0.05. The Mann-Whitney’s p value indicates the difference between the mean distances for the coagulated and non-coagulated nerves.
