# Supplementary material for: Development of an ultrasound-guided radiofrequency ablation technique in the equine cadaveric distal limb: histological findings and potential for treating chronic lameness
Source: Front Vet Sci. 2024 Aug 23;11:1437989. doi: 10.3389/fvets.2024.1437989 (PMC11377333; doi:10.3389/fvets.2024.1437989)
Supplement: Supplementary file 2 [file Table_2.docx]

Supplementary Material

**Supplementary Table 2.** Association between the tip-to-nerve ultrasound (US) distance (in mm) and the staining or the coagulation of non-target structures.

|  | | **Tip-to-nerve US distance (mm)** |
| --- | --- | --- |
| **Logistic estimates** | | *p* < 0.001 |
| **Other stained structures** | Artery † | 0 (0 – 1.6) |
|  | Vein ‡ | 2 (0 – 2.8) |
|  | Artery and vein † | 0.4 (0 – 1.2) |
|  | No † | 0 (0 – 0) |
| **Kruskal-Wallis** | | *p* = 0.002 |
| **Logistic estimates** | | *p* = 0.01 |
| **Other coagulated structures** | Artery | 0.4 (0.4 – 2.0) |
|  | Collagen | 0 (0 – 0) |
|  | Artery and collagen † | 0 (0 – 2.0) |
|  | No ‡ | 0 (0 – 2.8) |
| **Kruskal-Wallis** | | *p* = 0.002 |

‡ > † for *p* < 0.01. The Kruskal-wallis *p* value indicates the difference between the mean distances for the coagulated and non-coagulated nerves.
